# Hemodynamic Impact of Absent or Reverse End-Diastolic Flow in the Two Umbilical Arteries in Growth-Restricted Fetuses

**DOI:** 10.1371/journal.pone.0081160

**Published:** 2013-11-27

**Authors:** Edouard Lecarpentier, Anne Gaëlle Cordier, Francine Proulx, Jean Claude Fouron, Laurence Gitz, Gilles Grange, Alexandra Benachi, Vassilis Tsatsaris

**Affiliations:** 1 Obstetrics and Gynecology Unit, Maternité Port-Royal, APHP, Paris Descartes University, Paris, France; 2 Department of Obstetrics and Gynecology, Hôpital Antoine Béclère, Assistance Publique des Hôpitaux de Paris, Université Paris Sud, Paris, France; 3 Premup Foundation (Fondation pour la Prévention de la Prématurité et la Protection du Nouveau-né Prématuré), Paris, France; 4 Fetal Cardiology Unit, Pediatric Cardiology Service, Department of Pediatrics, Centre Hospitalier Universitaire (CHU) Saint-Justine, University of Montreal, Montreal, Canada; University of Tennessee Health Science Center, United States of America

## Abstract

**Objective:**

To determine if bilateral absent or reverse end-diastolic (ARED) flow in the two umbilical arteries (UAs) at the perivesical (PVC) segment represents a more severe degree of hemodynamic compromise than unilateral ARED flow at the PVC segment in singleton pregnancies complicated by intrauterine growth restriction (IUGR).

**Methods:**

This was a prospective observational study. One hundred nine fetuses with IUGR underwent a total of 225 ultrasound (US) examinations. We measured the pulsatility index (PI) from the two UAs at the PVC segment, UA in the free floating cord (FFC), middle cerebral artery (MCA), ductus venosus (DV) and the aortic isthmus blood flow index (IFI). Three groups were classified according to bilateral positive end-diastolic (PED) flow, unilateral ARED flow or bilateral ARED flow in the UAs at the PVC segment.

**Results:**

The proportions of US examinations with PED flow, unilateral ARED flow and bilateral ARED flow in the UAs were 54.7%, 20.4%, and 24.9%, respectively. At the last US examination, the IFI z-scores were significantly lower in the bilateral ARED group (-6.28±4.30) compared to the unilateral ARED group (-1.72±3.18, p<0.05) and the bilateral PED group (-0.83±2.36, p<0.05), the DV-PI z-scores were significantly higher in the bilateral ARED group (2.15±3.79) compared to the bilateral PED group (0.64±1.50, p<0.05). Before 32 weeks of gestation, the interval between US examination and delivery was significantly shorter in the bilateral ARED group (8.9 days ±8.2) than the unilateral ARED group (15.9 days ±13.4, p<0.05) and the bilateral PED group (30.3 days±25.7, p<0.05).

**Conclusion:**

There are significant differences in fetal blood fluxes between left and right UA. Doppler examination at the PVC segment significantly improves the comparability of UA-PI between two successive US examinations and allows a longitudinal and independent hemodynamic investigation of each UA. Examination of a single UA in free floating cord may miss a large fraction of unilateral ARED flow. In singleton IUGR fetuses, a bilateral ARED flow in the UAs at the PVC segment indicates more severe hemodynamic compromise and worse fetal conditions than unilateral ARED flow.

## Introduction

Fetuses with early-onset intrauterine growth restriction (IUGR) are at increased risk for adverse short- and long-term outcomes [1,2]. Obstetrical management consists in defining the optimal time of delivery and weighing the risks of prematurity against the risks of a potentially hostile intrauterine environment. A systematic review with meta-analysis has provided compelling evidence that perinatal morbidity and mortality can be reduced by using umbilical artery (UA) Doppler velocimetry to monitor placental perfusion in fetuses with early-onset IUGR [3]. UA Doppler assessment has therefore become a standard for antenatal management of IUGR. Recommendations on how to perform the exam have been published [4]. In particular, it was suggested that the UAs should be sampled in a free-floating loop of the umbilical cord (FFC), since the indices are higher when sampling occurs at the fetal end and lower at the placental end of the umbilical cord[5-11]. These differences are significant regardless of gestational age but do not seem to have an impact in clinical practice. In addition, the recommendations do not always include evaluation of both arteries, despite the fact that a more than 20% difference between them in Doppler indices has been reported in about a third of normal gestations [12]. Pulsatility index (PI) should therefore be measured on the same UA, especially for repeated measurements on the same fetus. The hemodynamic impact of the velocimetric differences between the 2 UAs has never been investigated. The main objective of the present study was to determine if bilateral absent or reverse end-diastolic (ARED) flow in the two UAs at the perivesical (PVC) segment represents a more severe degree of hemodynamic compromise than unilateral ARED flow at the PVC segment in singleton pregnancies complicated by intrauterine growth restriction (IUGR).

## Patients and Methods

### Study subjects

Between January 2010 and June 2011, a prospective cohort was created including 109 women with a singleton pregnancy complicated by IUGR of vascular origin. All fetuses were delivered in two Maternal-Fetal Medicine Departments: Maternité Port Royal (Université Paris-Descartes, Paris, France) and Hôpital Antoine Béclère (Université Paris Sud, Clamart, France).  Gestational age (GA) was defined as the time elapsed since 14 days prior to fertilization. IUGR was prenatally defined as an abdominal circumference in ultrasound below the 10^th^ percentile after checking the accuracy of the dating of the pregnancy with first trimester Crown-Rump Length, associated with abnormal Doppler findings at the uterine artery (unilateral or bilateral notching). Exclusion criteria were: congenital malformations, chromosomal abnormalities, multiple pregnancies and medical abortions. Newborns of birth weight above the 10^th^ percentile[13] were excluded (n=15).

### Ethics Statement

This non-interventional study was an evaluation of standard management at both hospitals and did not require any introduction of new diagnostic and therapeutic strategy (article L.1121-1-2°, décret 2006-477). Therefore, the ethics committee (Comité de Protection des Personnes Ile de France III) has specifically waived the requirement for approval. All US examinations were performed as part of the medical care of women and the requirement for written informed consent has also been waived by the ethics committee. All personal health information data were deidentified.

### Ultrasound and Doppler measurements

Two prenatal Doppler ultrasound examinations were performed every week in both centers by only one experienced operator (F.P.), using an Aloka ProSound Alpha 10 ultrasound system (Aloka, Wallingford, CT, USA) equipped with a 5 MHz linear curved-array transducer. All spectral Doppler measurements were performed automatically from five or more consecutive waveforms, with the angle of insonation as close to 0° as possible, in the absence of fetal movements and, if required, with voluntary maternal suspended breathing. The color flow mapping function was superimposed and the pulsed Doppler sample volume placed on the region of interest. The mechanical and thermal indices were maintained below 1. We measured Doppler waveforms from the UAs, middle cerebral artery (MCA), ductus venosus (DV) and aortic isthmus (AoI). PI was automatically calculated with peak systolic (PSV), end-diastolic (EDV) and time-averaged mean (TAMX) velocities (PI = [PSV-EDV]/TAMX) by the equipment software. UA-PI was routinely recorded bilaterally from the perivesical portion (less than 1 cm proximal to the abdominal insertion) and from a free-floating loop of the umbilical cord. MCA-PI was measured in a transverse section of the fetal head, as its proximal portion arising from the circle of Willis. DV-PI was measured in a mid-sagittal or transverse section of the fetal abdomen, positioning the Doppler gate at its isthmic portion. AoI Doppler waveforms were measured in a sagittal view of the fetal thorax with clear visualization of the aortic arch, placing the gate a few millimeters beyond the origin of the left subclavian artery. The aortic isthmus blood flow index (IFI) was obtained by dividing the sum of the systolic (VTI_S_) and diastolic (VTI_D_) Doppler blood flow velocity integrals by the systolic blood flow integral (IFI = (VTI_S_ + VTI_D_)/ VTI_S_)[14]. The cerebro-placental ratio (CPR) was calculated as the ratio of the MCA-PI to the FFC-UA-PI. The greater UA-PI value of the pair was designated UA-PI_max_ and the smaller UA-PI_min_. The percentage difference between UA-PI_max_ and UA-PI_min_ (∆UA-PI) was calculated for each pair of observations for each subject: ∆UA-PI = [( UA-PI_max_ - UA-PI_min_)/ UA-PI_min_] x 100. Using published normal values, the FFC-UA-PI[15], MCA-PI[16], CPR[16], IFI[14] and DV-PI[17] z-scores for gestational age (GA)was calculated, which allowed correction for the expected change in cerebral blood ﬂow that occurs across gestation. 

All deliveries were attended by a staff obstetrician. The sonographer was independent of the obstetrical teams and did not participate in the decision regarding fetal extraction. The obstetrician staff was aware of the Doppler findings recorded in the free floating cord site of the umbilical arteries as well as the other Doppler insonation sites, excluding PVC segment. 

### Statistical analysis

The Mann-Whitney nonparametric test and Pearson’s Chi square test were used to compare quantitative and qualitative data, respectively (*p* <0.05 was considered as significant). For multivarite analysis, variables with a p<0.05 were included in a logistic regression model. Data are expressed as odds ratio (OR) with 95% confidence interval (CI). P<0.05 was considered as significant. A linear regressions was used to establish a relationship between PVC-UA-PI max and PVC-UA-PI min. Statview 5.0 software, SAS Institute, was used for statistical analysis.

## Results

During the study period a total of 225 ultrasound (US) examinations were performed on 109 IUGR fetuses. A cesarean section was performed in 80 out of 109 pregnancies. The indications for elective cesarean section were abnormal fetal heart rate (n=47/80), absent or reverse flow in DV a-wave (n=5/80), fetal growth arrest (n=12/80), maternal complications secondary to preeclampsia (n=12/80), gestational age cut-off (absent end diastolic flow in the FFC after 34GA, n=4/80) (Table 1). The mean gestational ages at first/last US examinations were 29.2 wk±3.8 and 31.6wk±3.7, respectively (Table S1). The mean interval between the first and the last US examination was 4.4±4.6 weeks. The mean number of US examinations was 2.1±1.6. The proportion of cases with one, two or more than three examinations was 48.6%, 25.7% and 25.7%, respectively.

**Table 1:** Demographics, pregnancy, and management characteristics of the 109 women included in the study.

Maternal age (y, mean±SD)	31.0±6.2
Ethnic origin (#,%)	
White	66 (60.6)
Black	31 (28.4)
Other	12 (11)
Nulliparous (#,%)	71 (65.1)
Tobacco use (#,%)	23 (21.3)
Chronic hypertension (#,%)	18 (16.5)
Previous preeclampsia or IUGR (#,%)	16/37 (43.2)
Chronic renal disease or APS (#,%)	6 (5.5)
Aspirin (#,%)	14 (12.8)
Preeclampsia (#,%)	43 (39.5)
HELLP syndrome (#,%)	9 (8.3)
Mode of delivery	
Elective cesarean section (#,%)	80(73.4)
Cesarean section during labor (#,%)	4(3.7)
Vaginal delivery (#,%)	25(22.9)
Indication of elective cesarean delivery	
Non-reassuring FHR (#,%)	47/80 (58.8)
Venous Doppler (#,%)	5/80 (6.2)
Growth arrest (#,%)	12/80 (15)
Maternal (#,%)	12/80 (15)
Gestational age cut-off (#,%)	4/80 (5)

SD: standard deviation, IUGR: intrauterine growth restriction, APS: antiphospholipid syndrome, HELLP: hemolytic anemia, elevated liver enzymes and low platelet count, FHR: fetal heart rate.

**Figure 1:**
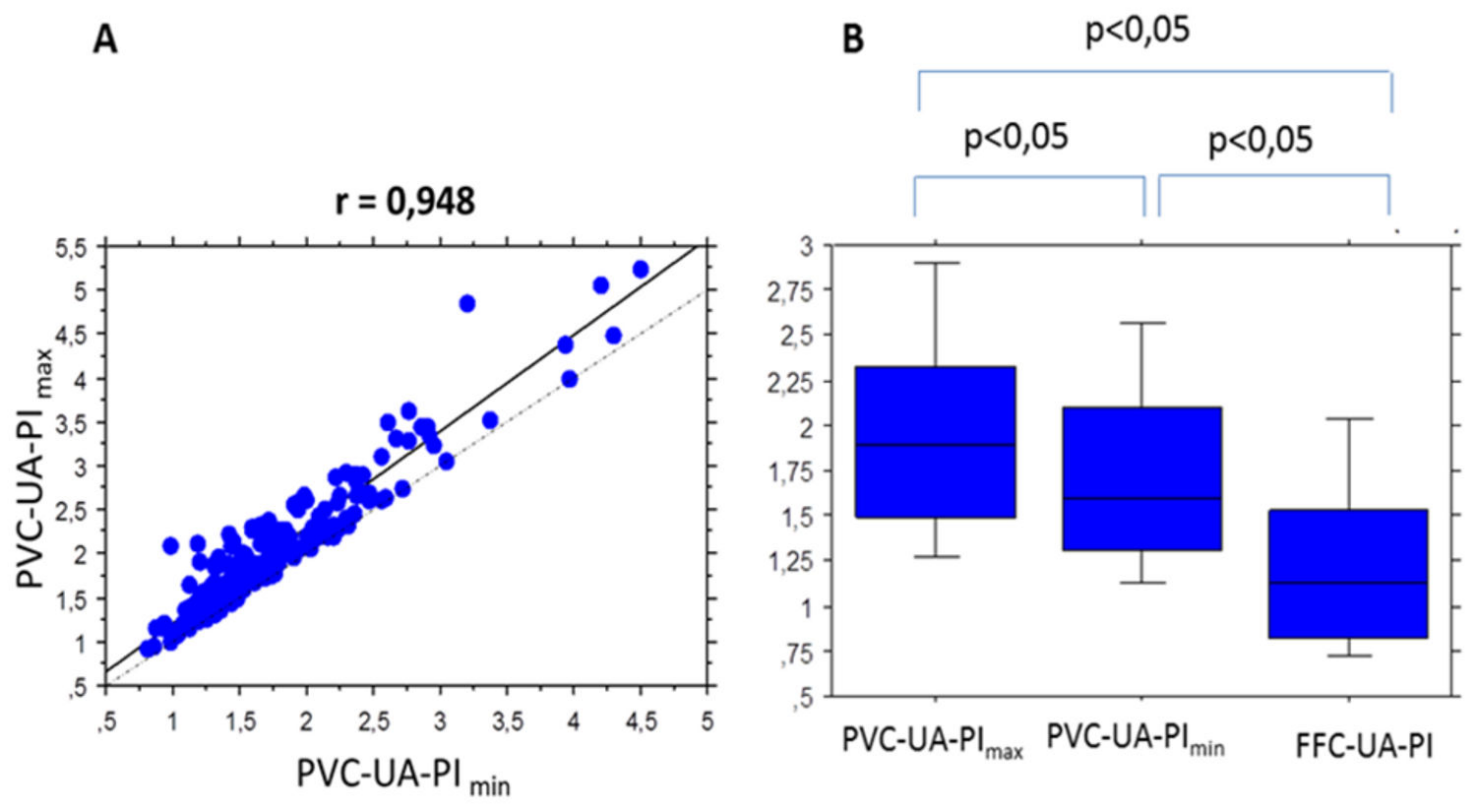
A: Correlation between the two UA-PIs measured at the perivesical (PVC) segment. The larger UA-PI value of the pair was designated PVC-UA-PI_max_ and the smaller, PVC-UA-PI_min_. The dotted line depicts the line of equality. B: Comparison between the UA-PI measured at the PVC segment and in the free-floating cord (FFC).

The overall mean (±standard deviation SD) UA-PI_max_ was 2.05±0.76; and the overall mean (±SD) UA-PI_min_ was 1.77±0.66. The difference of 0.28±0.25 was statistically significant (*p*<0.0001). The mean (±SD) percent difference (∆UA-PI) was 16.7±15.1%. However, there was a good correlation between the UA-PI_max_ and the UA-PI_min_ (r= 0.948) (Figure 1A). In the FFC, the overall mean (±SD) UA-PI was 1.30±0.75, which is significantly less than between the UA-PI_max_ (*p*<0.0001) and the UA-PI_min_ (*p*<0.0001) measured at the PVC segment (Figure 1B).

The proportion of fetuses with at least once an absent or reverse end-diastolic (ARED) flow in the UAs was 50.5%. The proportions of US examinations with a positive end-diastolic (PED) flow in the UAs (Bilateral PED group n=123), a unilateral ARED flow in the UAs (Unilateral ARED group n=46) and a bilateral ARED flow in the UAs (Bilateral ARED group n=56) were 54.7%, 20.4%, and 24.9%, respectively. The overall perinatal death rate was 8/109 (7.3%) (Table 2); 6 of these 8 fetuses had at least once a bilateral ARED flow in the UAs. 

**Table 2:** Perinatal outcomes.

Delivery GA (wk, mean±SD)	33.3±4.2
Delivery GA	
<26 weeks (#,%)	1 (1)
[26-32[ weeks (#,%)	39 (35.8)
[32-37[ weeks (#,%)	38 (34.8)
GA >37 weeks (#,%)	31 (28.4)
Birth weight (g, mean±SD)	1435±680
Birth weight <5^th^ centile (#,%)	70 (64.2)
1 min Apgar score < 7 (%)	18/105 (17.3)
5 min Apgar score < 7 (%)	5/105 (4.8)
10 min Apgar score < 7 (%)	1/105 (1)
Umbilical arterial blood pH (mean±SD)	7.29±0.1
Admission to neonatal care (%)	62/105 (60)
IUFD (#,%)	4/109 (3.7)
Neonatal death (#,%)	2/105 (1.9)

GA: gestational age, IUFD: intrauterine fetal death, Perc: percentile

Different numbers of US examinations were performed in each IUGR case. The analysis shown in the table S2 and table 3 only take into account the measurement at the first and last US examination for each case respectively. 21 (19.3%) women had a bilateral ARED flow in the PVC-UAs at the first US examination (Table S2) versus 36 (33%) at the last US examination (Table 3). The UA-PI z-scores in the FFC at the last US examination were significantly higher in the bilateral ARED group (4.26±5.74) compared to the unilateral ARED group (2.23±6.70) and the bilateral PED group (0.14±1.6). The MCA-PI z-scores at the last US examination were significantly lower in the bilateral ARED group (-1.93±0.57) compared to the unilateral ARED group (-1.79±0.65) and the bilateral PED group (-1.12±0.96). The CPR z-scores at the last US examination were significantly lower in the bilateral ARED group (-3.02±1.15) compared to the unilateral ARED group (-2.25±1.11) and the bilateral PED group (-0.95±1.72). The IFI z-scores at the last US examination were significantly lower in the bilateral ARED group (-6.28±4.30) compared to the unilateral ARED group (-1.72±3.18) and the bilateral PED group (-0.83±2.36). The DV-PI z-scores at the last US examination were significantly higher in the bilateral ARED group (2.15±3.79) compared to the bilateral PED group (0.64±1.50). Considering only the last US examination for each patient, in univariate analysis, the FFC-UA-PI z-score, the CPR z-score and the IFI z-score were significantly associated with a bilateral ARED flow in the umbilical artery at the PVC segment. In multivariate analysis, only the IFI z-score was independently and significantly associated with a bilateral ARED flow in the umbilical artery at the PVC segment (OR=1.56, CI95% 1.02-2.37, p<0.05).

**Table 3:** Gestational age at the last US examination, interval between the last US examination and delivery, general hemodynamic characteristics depending on the degree of placental compromise at the last US examination (n=109).

	Bilateral PED (n=54)	Unilateral ARED (n=19)	Bilateral ARED (n=36)
GA at US examination (wk, mean±SD)	32.9±3.8	30.3±2.8*	29.6±2.9*
Interval between US examination and delivery (days, mean±SD)	16.8±22.9	7.1±10.8	4.5±5.1*
FFC-UA-PI (mean±SD)	0.94±0.29	1.42±1.40*	1.84±1.22*
FFC-UA-PI z-score (mean±SD)	0.14±1.6	2.23±6.70*	4.26±5.74*†
MCA-PI (mean±SD)	1.53±0.41	1.33±0.30*	1.27±0.25*
MCA-PI z-score (mean±SD)	-1.12±0.96	-1.79±0.65*	-1.93±0.57*
CPR (mean±SD)	1.79±0.72	1.26±0.49*	0.92±0.50*†
CPR z-score (mean±SD)	-0.95±1.72	-2.25±1.11*	-3.02±1.15*†
CPR < 1 (#,%)	8/52 (15.4)	3/14 (21.4)*	16/25 (64.0)*
IFI (mean±SD)	1.16±0.29	1.08±0.37*	0.64±0.42*†
IFI z-score (mean±SD)	-0.83±2.36	-1.72±3.18	-6.28±4.30*†
DV-PI (mean±SD)	0.62±0.23	0.61±0.16	0.88±0.60*
DV-PI z-score (mean±SD)	0.64±1.50	0.49±1.01	2.15±3.79*

GA: gestational age. PED: positive end diastolic flow in the umbilical artery at the PVC segment, ARED: absent or reverse flow in the umbilical artery at the PVC segment, CPR: cerebro-placental ratio, IFI: aortic isthmus blood flow index, DV: Ductus Venosus, PI: pulsatility index. *: significant difference (p<0.05) with the PED group. †: significant difference (p<0.05) with the unilateral ARED group.

Among the UA Doppler examinations performed before 32 weeks of gestation (n=129) (Table 4), interval before delivery was significantly shorter in the bilateral ARED group (8.9 days±8.2) than in the unilateral ARED group (15.9 days±13.4) and the bilateral PED group (30.3 days±25.7). There were no significant differences of gestational age at US examination between the three groups. The UA-PI z-scores in the FFC before 32 weeks of gestation were significantly higher in the bilateral ARED group (3.66±5.49) compared to the unilateral ARED group (2.17±5.59) and the bilateral PED group (0.29±1.81). The CPR z-scores before 32 weeks of gestation were significantly lower in the bilateral ARED group (-2.64±1.34) compared to the unilateral ARED group (-1.92±1.39) and the bilateral PED group (-0.99±1.39). The IFI z-scores at the last US examination were significantly lower in the bilateral ARED group (-4.21±3.87) compared to the unilateral ARED group (-2.14±3.05) and the bilateral PED group (-0.09±1.61). The DV-PI z-scores at the last US examination were significantly higher in the bilateral ARED group (1.65±3.16) compared to the bilateral PED group (0.50±1.91).

**Table 4:** Gestational age at US examination, interval between US examination and delivery, general hemodynamic characteristics depending on the degree of placental compromise.

	Bilateral PED (n=57)	Unilateral ARED (n=27)	Bilateral ARED (n=45)
GA at US examination (wk, mean±SD)	28.8±2.01	27.9±2.9	28.5±2.1
Interval between US examination and delivery (days, mean±SD)	30.3±25.7	15.9±13.4*	8.9±8.2*†
FFC-UA-PI (mean±SD)	1.06±0.36	1.45±1.16*	1.75±1.09†
FFC-UA-PI z-score (mean±SD)	0.29±1.81	2.17±5.59*	3.66±5.49*†
MCA-PI (mean±SD)	1.70±0.4	1.46±0.35*	1.38±0.35*
MCA-PI z-score (mean±SD)	-0.85±0.94	-1.34±0.88*	-1.65±0.76*
CPR (mean±SD)	1.22±0.38	0.73±0.25*	0.54±0.21*†
CPR z-score (mean±SD)	-0.99±1.39	-1.92±1.39*	-2.64±1.34*†
CPR < 1 (#,%)	3/50 (6.0)	6/22 (27.3)*	22/41(53.7)*
IFI (mean±SD)	1.27±0.15	1.11±0.23*	0.90±0.33*†
IFI z-score (mean±SD)	-0.09±1.61	-2.14±3.05*	-4.21±3.87*†
DV-PI (mean±SD)	0.63±0.3	0.70±0.17*	0.81±0.41*
DV-PI z-score (mean±SD)	0.50±1.91	0.91±1.10*	1.65±3.16*

US examinations performed before 32 weeks of gestation (n=129).

GA: gestational age. PED: positive end diastolic flow in the umbilical artery at the PVC segment, ARED: absent or reverse flow in the umbilical artery at the PVC segment, CPR: cerebro-placental ratio, IFI: aortic isthmus blood flow index, DV: Ductus Venosus, PI: pulsatility index. *: significant difference (p<0.05) with the PED group. †: significant difference (p<0.05) with the unilateral ARED group.

The birth weight and the gestational age at birth were significantly higher in fetuses with no ARED flow in the UAs (n=54) compared with fetuses with at least once a unilateral ARED flow (n=19) and a bilateral ARED flow (n=36) in the UAs (Table 5).

**Table 5:** Perinatal outcomes depending on the degree of placental compromise.

	Bilateral PED (n=54)	Unilateral ARED (n=19)	Bilateral ARED (n=36)
Birth weight (g, mean±SD)	1844±663	1044±411*	1027±402*
Delivery GA (wk, mean±SD)	35.8±3.6	31.3±3.2*	31.2±2.7*
1 min Apgar score (mean±SD)	8.9±1.5	7.2±2.8*	7.4±2.6*
5 min Apgar score (mean±SD)	9.5±1.0	8.5±2.6*	9.6±1.4*
10 min Apgar score (mean±SD)	9.8±0.5	9.3±1.9	9.6±0.7
Umbilical arterial blood pH (mean±SD)	7.3±0.1	7.3±0.1	7.3±0.1

PED: positive end diastolic flow in the umbilical artery, ARED: absent or reverse flow in the umbilical artery, CPR: cerebro-placental ratio, IFI: aortic isthmus blood flow index, PI: pulsatility index. *: significant difference (p<0.05) with the PED group.

## Discussion

Our results show that in growth-restricted fetuses the two UAs do not have a similar impedance to flow. From 225 US examinations, an ARED flow at the PVC segment was observed in 45.3% (n=102) of cases. Among those, the ARED flow concerned both UAs in 55% (n=56) of cases and only one UA in 45% (n=46) of cases. If the Doppler measurements had been made on a single UA in the FFC, 22.5% of cases of ARED flow (half of the 46 cases of unilateral ARED flow) would have been unnoticed, which represents 10% of all US examinations. This observation does not take into account the fact that, in the same UA, an ARED flow at the PVC segment can correspond with a PED flow in the FFC. Moreover, it should be noted that, among the 225 US examinations, only 54.9% bilateral ARED flow at the PVC segment were associated with a UA-PI > 95^th^ Percentile in the FFC. 

Different numbers of US examinations were performed in each IUGR case. This may lead to a bias since the earliest and most severe cases could account for more examinations than other cases, therefore we analyzed the results of the first (Table S2) and last US examinations (Table 3).

IUGR fetuses have an acid-base imbalance compared with fetuses without growth restriction at the same gestational age[18]. Fetuses with decreased oxygen delivery due to placental dysfunction display cerebral vasodilation enhancing cerebral perfusion as an adaptive phenomenon. By studying cordocentesis in 19 fetuses thought to be small-for-gestational age at 28-35 weeks of gestation, Bonnin et al have demonstrated that umbilical venous blood gases could be related to umbilical circulatory function[19]. Fetal hypoxia, hypercapnia and acidosis can be related to an altered umbilical vascular network. Rizzo et al[20] showed that the PI of the MCA is the indicator that best predicts fetal hypoxemia. Our results show a non-significant trend toward a greater cerebral vasodilation in the bilateral ARED group than in the unilateral ARED group, which suggests that the fetal hypoxemia is probably greater in the bilateral ARED group. The neonatal umbilical arterial blood pH is not significant different between the three groups: PED, unilateral ARED and bilateral ARED (Table 5). Unfortunately it is not possible to establish any correlation between fetal Doppler findings and cord blood gases because on the one hand these measures were not obtained at the same time and on the other hand only one UA was sampled for blood gases.

The combination of both an increase in resistance in the umbilical circulation and a fall in cerebral vascular resistance observed in vascular IUGR is responsible for the diastolic flow patterns observed in the aortic isthmus and the changes in IFI[21]. In our study, the IFI values were significantly lower in the bilateral ARED group (mean value <1) than in the unilateral ARED group (mean value > 1). These results demonstrate a more severe placental circulatory insufficiency and a greater right ventricular afterload in the bilateral ARED flow group.

Doppler examination of the DV plays an important role in the management of fetuses with IUGR [22,23]. An increased pulsatility of the DV blood velocity is associated with fetal acidosis and hypoxemia[20]. The DV-PI, which reflects the impaired myocardial relaxation and the reduced compliance of the distended venous system, was significantly higher in the bilateral ARED flow group, including US examinations conducted before 32 weeks of gestation. This suggests that the bilateral ARED flow pattern reflects a more advanced hemodynamic compromise. 

Spinillo et al founded that in 72 normotensive pregnancies complicated of IUGR, abnormalities of UA Doppler velocimetry were associated with increased intervillous ﬁbrin deposits, villous hypoplasia, syncytial knots, placental site giant cells, immature intermediate trophoblast, and with pattern of lesions indicating superﬁcial implantation and maternal vascular underperfusion[24]. Our results reinforce these observations. Considering only the examinations conducted before 32 weeks of gestation, the mean gestational age was not significantly different between the three groups, and the average time between the examination and the decision to extract the fetus was significantly shorter in the bilateral ARED group than in the unilateral ARED group and the bilateral PED group. The results of UA Doppler measured at the PVC segment were not taken into account by the medical teams who made the decision regarding fetal extraction. This suggests that bilateral ARED flow in UAs at the PVC segment reveals an altered placental perfusion which compromises fetal growth and fetal well-being more severely than unilateral ARED flow. 

Janeczek et al have shown that the laterality of the UA, whether on the left or right side of the fetus, influences the Doppler blood flow parameters[25]. In their study, most fetuses had a significantly higher PI in the left umbilical artery (1.18 vs 1.12, p=0.037, n=246). The difference was not significant in the IUGR group (1.19 vs 1.11, p=0.3, n=16). In our study, we did not find that the PI at the PVC segment was significantly higher in the left UA (1.91±0.86 vs 1.91±0.72, p=0.77).

Balbis et al have shown that measurements of the UAs sampled at the PVC segment could be biased by the degree of curvature of the vessel due to bladder filling and should only be performed when the fetal bladder is empty[26]. The degree of filling of the bladder at the time of measurement was not taken into account in our study; it can be assumed, however, that this potential confounding variable has a similar effect on both arteries and should not have a significant influence on the PI differences. 

The Hyrtl anastomosis is a communication between the two UAs near the surface of the placenta. It seems to play a physiological role in the equalization of flow and pressures between the two arteries for the uniform distribution of blood to the different lobes of the placenta. Predanic et al[12] have postulated that failure of the Hyrtl anastomosis to develop anatomically or to function fully may result in differences in flow patterns between the two UAs. Ullberg et al found that the Hyrtl anastomosis in placentas from IUGR had a varied anatomy and a relationship between its width and the symmetry of the supply areas of each umbilical artery[27]. Their anatomopathological results did not differ between placentas from IUGR and placentas from normal pregnancies, suggesting that the Hyrtl anastomosis is not involved in the pathogenesis of IUGR. The good correlation between the UA-PI_max_ and the UA-PI_min_ (r= 0.948) may be related to altered vascular networks downstream from one umbilical artery compare to the other, whenever the Hyrtl anastomose is patent or not. Flows in the Hyrtl anastomosis in the case of discrepancy between the two UAs could be the subject of a future study.

A major limitation of our study is that we have not measured systematically the velocities in both UA in the FFC. For the same UA, an ARED flow in the FFC supposes an ARED flow at the PVC segment, but the reverse is not true. Our results show that at the PVC segment, a bilateral ARED flow entails a more compromised fetal hemodynamic status than an unilateral ARED flow. It is therefore possible to deduce that in the FFC, a bilateral ARED flow entails probably a more compromised fetal hemodynamic status. 

Our results describe the hemodynamic status for each fetus at a given moment, in order to describe the relationship between the two UAs at the PVC segment and three other sites: the middle cerebral artery, aortic isthmus and ductus venosus. Further studies are needed to clarify the place of bilateral ARED flow in the temporal sequence of hemodynamic changes in severe IUGR in singleton pregnancies. 

## Conclusion

There are significant differences in fetal blood fluxes between left and right UA. Doppler examination at the PVC segment significantly improves the comparability of UA-PI between two successive US examinations and allows a longitudinal and independent hemodynamic investigation of each UA. Examination of a single UA in the FFC may miss a large fraction of unilateral ARED flow. In singleton IUGR fetuses, a bilateral ARED flow in the UAs at the PVC segment indicates more severe hemodynamic compromise and worse fetal conditions than unilateral ARED flow. Further studies are needed to clarify the place of bilateral ARED flow both in the temporal sequence of hemodynamic changes and in the timing of delivery of severe IUGR singleton pregnancies, taking into account perinatal mortality and morbidity, including postnatal neurodevelopment.

## Supporting Information

Table S1 **Gestational age, interval between US examination and delivery, hemodynamic characteristics depending on the degree of placental compromise. US examinations (n=225).**
(DOCX)

Table S2 **Gestational age at the first US examination, interval between the first US examination and delivery, hemodynamic characteristics depending on the degree of placental compromise at the first US examination (n=109).**
(DOCX)
