# Supplementary material for: Hemodynamic Impact of Absent or Reverse End-Diastolic Flow in the Two Umbilical Arteries in Growth-Restricted Fetuses
Source: PLoS One. 2013 Nov 27;8(11):e81160. doi: 10.1371/journal.pone.0081160 (PMC3842244; doi:10.1371/journal.pone.0081160)
Supplement: Table S1 — Gestational age, interval between US examination and delivery, hemodynamic characteristics depending on the degree of placental compromise. US examinations (n=225). (DOCX) [file pone.0081160.s001.docx]

Table S1: Gestational age, interval between US examination and delivery, hemodynamic characteristics depending on the degree of placental compromise. US examinations (n=225).

|  | Bilateral  PED  (n=123) | Unilateral  ARED  (n=46) | Bilateral  ARED  (n=56) |
| --- | --- | --- | --- |
| GA at US examination (wk, mean±SD) | 32.0±3.6 | 30.1±3.5* | 29.5±2.8* |
| Interval between US examination and delivery (days, mean±SD) | 20.4±21.0 | 13.4±11.6 | 7.8±7.8*† |
| FFC-UA-PI (mean±SD) | 1.05±0.33 | 1.43±0.92* | 1.71±0.99*† |
| FFC-UA-PI z-score (mean±SD) | 0.61±1.68 | 2.33±4.43* | 3.58±4.72* |
| MCA-PI (mean±SD) | 1.61±0.43 | 1.41±0.31* | 1.36±0.31* |
| MCA-PI z-score (mean±SD) | -1.02±0.98 | -1.53±0.79* | -1.70±0.73* |
| CPR (mean±SD) | 1.65±0.64 | 1.20±0.49* | 1.02±0.52*† |
| CPR z-score (mean±SD) | -1.32±1.55 | -2.37±1.28* | -2.77±1.28* |
| CPR < 1 (#,%) | 13/101 (12.9) | 15/40 (37.5)* | 31/51 (60.8)*† |
| IFI (mean±SD) | 1.21±0.27 | 1.04±0.34* | 0.83±0.38*† |
| IFI z-score (mean±SD) | -0.45±2.17 | -2.30±3.26* | -4.45±3.86*† |
| DV-PI (mean±SD) | 0.60±0.25 | 0.65±0.17* | 0.70±0.23* |
| DV-PI z-score (mean±SD) | 0.50±1.59 | 0.70±1.09 | 1.50±2.90* |

GA: gestational age. PED: positive end diastolic flow in the umbilical artery at the PVC segment, ARED: absent or reverse flow in the umbilical artery at the PVC segment, CPR: cerebro-placental ratio, IFI: aortic isthmus blood flow index, DV: Ductus Venosus, PI: pulsatility index. *: significant difference (p<0.05) with the PED group. †: significant difference (p<0.05) with the unilateral ARED group.
