# Supplementary material for: Hemodynamic Impact of Absent or Reverse End-Diastolic Flow in the Two Umbilical Arteries in Growth-Restricted Fetuses
Source: PLoS One. 2013 Nov 27;8(11):e81160. doi: 10.1371/journal.pone.0081160 (PMC3842244; doi:10.1371/journal.pone.0081160)
Supplement: Table S2 — Gestational age at the first US examination, interval between the first US examination and delivery, hemodynamic characteristics depending on the degree of placental compromise at the first US examination (n=109). (DOCX) [file pone.0081160.s002.docx]

Table S2: Gestational age at the first US examination, interval between the first US examination and delivery, hemodynamic characteristics depending on the degree of placental compromise at the first US examination (n=109).

|  | Bilateral PED (n=67) | Unilateral ARED (n=21) | Bilateral ARED (n=21) |
| --- | --- | --- | --- |
| GA at US examination (wk, mean±SD) | 31.6±3.8 | 29.6±3.5* | 28.2±2.2* |
| Interval between US examination and delivery (days, mean±SD) | 25.1±23.4 | 10.2±9.8* | 10.1±9.4* |
| FFC-UA-PI (mean±SD) | 0.97±0.31 | 1.32±1.23* | 1.87±1.37* |
| FFC-UA-PI z-score (mean±SD) | 0.20±1.57 | 1.70±5.95* | 4.15±6.47* |
| MCA-PI (mean±SD) | 1.63±0.40 | 1.35±0.30* | 1.35±0.32* |
| MCA-PI z-score (mean±SD) | -0.95±0.93 | -1.69±0.86* | -1.71±0.71* |
| CPR (mean±SD) | 1.75±0.57 | 1.33±0.56* | 1.05±0.60* |
| CPR z-score (mean±SD) | -1.03±1.39 | -1.97±1.47* | -2.57±1.40* |
| CPR < 1 (#,%) | 5/67 (7.5) | 5/21 (23.8) | 12/21 (57.1)* |
| IFI (mean±SD) | 1.28±0.16 | 1.00±0.33* | 0.99±0.30* |
| IFI z-score (mean±SD) | 0.02±1.6 | -2.62±3.07* | -3.35±3.94* |
| DV-PI (mean±SD) | 0.58±0.28 | 0.63±0.20 | 0.90±0.69* |
| DV-PI z-score (mean±SD) | 0.34±1.75 | 0.57±1.28 | 2.22±4.39* |

GA: gestational age. PED: positive end diastolic flow in the umbilical artery at the PVC segment, ARED: absent or reverse flow in the umbilical artery at the PVC segment, CPR: cerebro-placental ratio, IFI: aortic isthmus blood flow index, DV: Ductus Venosus, PI: pulsatility index. *: significant difference (p<0.05) with the PED group. †: significant difference (p<0.05) with the unilateral ARED group.
